# Ethyl 27-oxo-15-oxa-2,20-diaza­hexa­cyclo­[18.6.1.0^1,8^.0^2,6^.0^9,14^.0^21,26^]hepta­cosa-9,11,13,21,23,25-hexa­ene-7-carboxyl­ate

**DOI:** 10.1107/S1600536812049082

**Published:** 2012-12-05

**Authors:** Sibi Narayanan, Thothadri Srinivasan, Santhanagopalan Purushothaman, Raghavachary Raghunathan, Devadasan Velmurugan

**Affiliations:** a Centre of Advanced Study in Crystallography and Biophysics, University of Madras, Guindy Campus, Chennai 600 025, India; b Department of Organic Chemistry, University of Madras, Guindy Campus, Chennai 600 025, India

## Abstract

In the title compound, C_27_H_30_N_2_O_4_, the pyrrolidine ring adopts a twisted conformation. The indoline ring system is almost perpendicular to the mean plane of the pyrrolidine ring, making a dihedral angle of 81.7 (8)°. In the crystal, mol­ecules are linked into centrosymmetric dimers with graph-set motif *R*_2_^2^(16) *via* pairs of C—H⋯O hydrogen bonds. The terminal ethyl group of the ester group is disordered over two sets of sites, with a site-occupancy ratio of 0.587 (11):0.413 (11).

## Related literature

For the biological activity of spiro-pyrrolidine derivatives, see: Obniska *et al.* (2003 ▶); Peddi *et al.* (2004 ▶); Christoph *et al.* (2011 ▶); Stylianakis *et al.* (2003 ▶); Waldmann (1995 ▶); Suzuki *et al.* (1994 ▶); Huryn *et al.* (1991 ▶). For a related structure, see: Ganesh *et al.* (2012 ▶). For puckering parameters, see: Cremer & Pople (1975 ▶) and for asymmetry parameters, see: Nardelli *et al.* (1983 ▶).
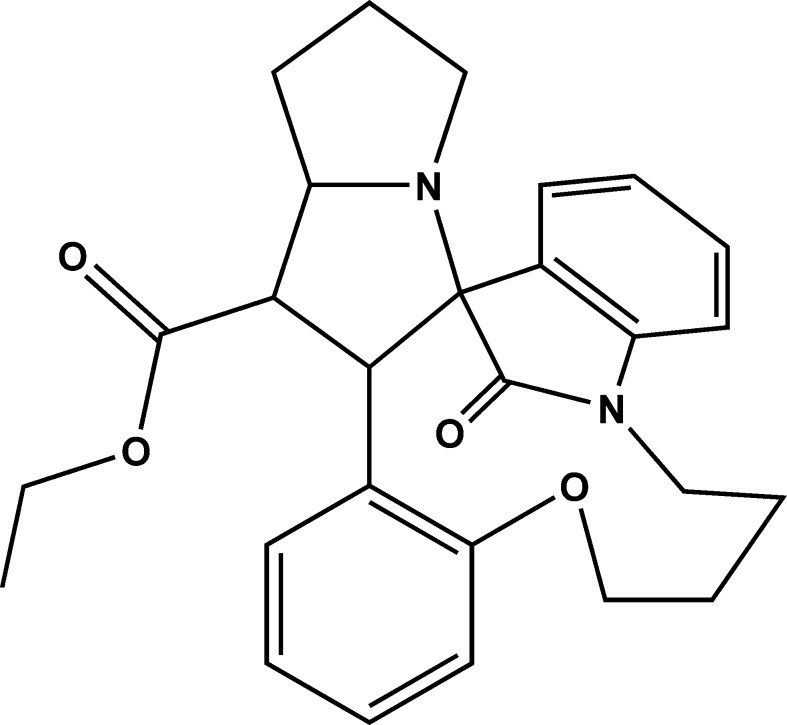


## Experimental

### 

#### Crystal data

C_27_H_30_N_2_O_4_
*M* *_r_* = 446.53
Triclinic, 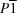

*a* = 8.9327 (5) Å
*b* = 10.0068 (5) Å
*c* = 14.6379 (11) Å
α = 103.988 (4)°
β = 95.023 (4)°
γ = 113.775 (3)°
*V* = 1136.41 (13) Å^3^
*Z* = 2
Mo *K*α radiation
μ = 0.09 mm^−1^
*T* = 293 K
0.30 × 0.30 × 0.25 mm

#### Data collection

Bruker APEXII CCD area-detector diffractometer
Absorption correction: multi-scan (*SADABS*; Bruker, 2008 ▶) *T*_min_ = 0.974, *T*_max_ = 0.978
20472 measured reflections
5603 independent reflections
4378 reflections with *I* > 2σ(*I*)
*R*_int_ = 0.025

#### Refinement

*R*[*F* ^2^ > 2σ(*F* ^2^)] = 0.045
*wR*(*F* ^2^) = 0.125
*S* = 1.08
5603 reflections
319 parameters
40 restraints
H-atom parameters constrained
Δρ_max_ = 0.23 e Å^−3^
Δρ_min_ = −0.22 e Å^−3^

### 

Data collection: *APEX2* (Bruker, 2008 ▶); cell refinement: *SAINT* (Bruker, 2008 ▶); data reduction: *SAINT*; program(s) used to solve structure: *SHELXS97* (Sheldrick, 2008 ▶); program(s) used to refine structure: *SHELXL97* (Sheldrick, 2008 ▶); molecular graphics: *ORTEP-3* (Farrugia, 2012 ▶); software used to prepare material for publication: *SHELXL97* and *PLATON* (Spek, 2009 ▶).

## Supplementary Material

Crystal structure: contains datablock(s) global, I. DOI: 10.1107/S1600536812049082/bt6853sup1.cif

Structure factors: contains datablock(s) I. DOI: 10.1107/S1600536812049082/bt6853Isup2.hkl

Supplementary material file. DOI: 10.1107/S1600536812049082/bt6853Isup3.cml

Additional supplementary materials:  crystallographic information; 3D view; checkCIF report

## Figures and Tables

**Table 1:** Hydrogen-bond geometry (Å, °)

*D*—H⋯*A*	*D*—H	H⋯*A*	*D*⋯*A*	*D*—H⋯*A*
C11—H11⋯O1^i^	0.93	2.49	3.3957 (19)	164
C12—H12⋯O2^ii^	0.93	2.59	3.446 (2)	153
C13—H13⋯O4^ii^	0.93	2.47	3.3986 (17)	175

Symmetry codes: (i) 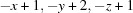; (ii) 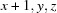.

**Fig. 1.**
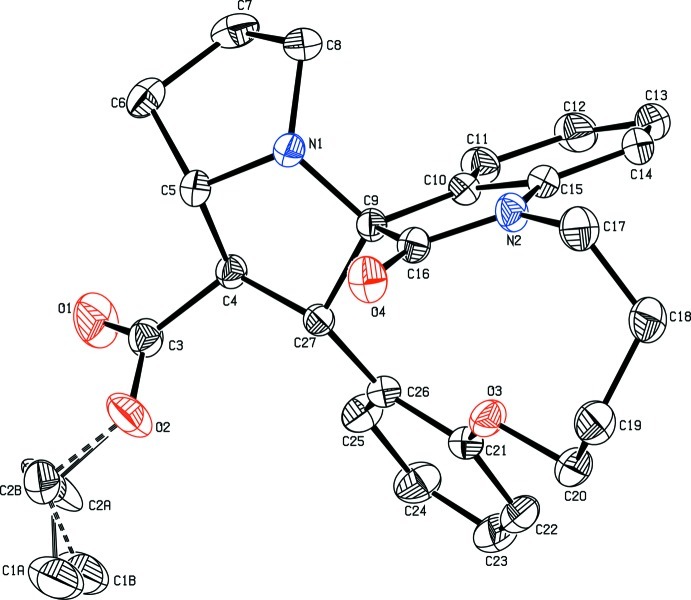
The molecular structure of the title compound, showing displacement ellipsoids drawn at the 30% probability level. H atoms are omitted for figure clarity.

**Fig. 2.**
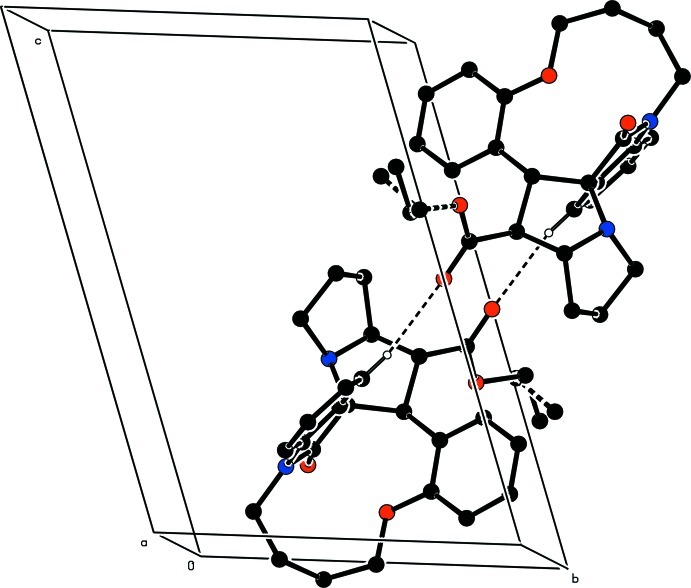
The crystal structure showing the formation of the centrosymmetric R22(16) dimer. H atoms are not involed in hydrogen bonds have been omitted.

## supplementary crystallographic information

### Comment

Spiro-pyrrolidine derivatives are unique tetracyclic 5-HT(2 A) receptor antagonists (Obniska *et al.*, 2003; Peddi *et al.*, 2004). These derivatives possess anticancer (Christoph *et al.*, 2011) and anti-influenza virus (Stylianakis *et al.*, 2003) activities. Highly functionalized pyrrolidines have gained much interest in the past few years as they constitute the main structural element of many natural and synthetic pharmacologically active compounds (Waldmann, 1995). Optically active pyrrolidines have been used as intermediates, chiral ligands or auxiliaries in controlled asymmetric synthesis (Suzuki *et al.*, 1994; Huryn *et al.*, 1991). In view of these importance and in continuation of our work on the crystal structure analysis of spiro-pyrrolidine derivatives, the crystal structure of the title compound has been carried out and the results are presented here.

X-Ray analysis confirms the molecular structure and atom connectivity as illustrated in Fig. 1. The bond lengths and angles are within normal ranges and comparable to those found in related structures (Ganesh *et al.*, 2012). Terminal atoms C1 & C2 is substituted at propanate group, which is disordered over two positions [C1A/C1B & C2A/C2B] with a site-occupancy ratio of 0.604 (5):0.396 (5). The sum of the angles at N1 & N2 [339 (1)° & 348.7 (1)°] of the pyrrolidine and indole rings are in accordance with *sp^3^* hybridization.

The indoline ring system is essentially planar, with maximum deviation of 0.038 (2) Å for atom C16. The pyrrolidine ring system makes dihedral angle of 81.7 (8)° with the indoline ring system, it clearly shows that both the rings are perpendicular to each other. The propanate group assumes an extended conformation which can be seen from the torsion angle [C4/C3/O2/C2= 179.3 (2)°].

The dihedral angle of the pyrrolidine ring and the benzene ring (C21—C26) is 36.5 (1)°. The atom O4 deviates by 0.102 (1) Å from the leastsquares plane of the indole ring. The pyrrolidine ring adopts *twisted* conformation [it is twisted about C1—C2], with the puckering parameters q_2_ and φ (Cremer & Pople, 1975) and the smallest displacement asymmetric parameter, Δ_s_, (Nardelli *et al.*, 1983) as follows: q_2_=0.4071 (2) Å, φ=82.0 (8)°, Δ_s_(C4)=7.61 (2). The crystal packing is stabilized by C—H···O interactions.

### Experimental

A solution of (*E*)-ethyl 3-(2-(4-(2,3-dioxoindolin-1-yl)butoxy)phenyl)acrylate (200 mg, 0.5 mmol) and *L*-proline (70 mg, 0.61 mmol) was refluxed in dry toluene under N_2_ atmosphere for 8hrs under Dean-Stark apparatus. After the completion of reaction as indicated by TLC, toluene was evaporated under reduced pressure. The crude product was washed with water and extracted with dichloromethane (4x20mL). The combined organic layers were dried (MgSO_4_) and filtered, concentrated in vacuum. The crude product was purified by column chromatography using hexane: EtOAc (8:2) as eluent.

### Refinement

Atoms C1 and C2 are disordered over two positions (C1A/C1B & C2A/C2B) with refined occupancies of 0.587 (11):0.413 (11). The O-C and C-C distances of the disordered atoms were restrained to be equal. The displacement parameters of the disordered atoms were restrained to be equal for bonded atoms. All H atoms were fixed geometrically and allowed to ride on their parent C atoms, with C—H distances fixed in the range 0.93–0.97 Å with *U*_iso_(H) = 1.5*U*_eq_(C) for methyl H, 1.2*U*_eq_(C) for other H atoms.

### Crystal data

**Table:**

C_27_H_30_N_2_O_4_	*Z* = 2
*M**_r_* = 446.53	*F*(000) = 476
Triclinic, *P*1	*D*_x_ = 1.305 Mg m^−^^3^
Hall symbol: -P 1	Mo *K*α radiation, λ = 0.71073 Å
*a* = 8.9327 (5) Å	Cell parameters from 5603 reflections
*b* = 10.0068 (5) Å	θ = 1.5–28.3°
*c* = 14.6379 (11) Å	µ = 0.09 mm^−^^1^
α = 103.988 (4)°	*T* = 293 K
β = 95.023 (4)°	Block, white crystalline
γ = 113.775 (3)°	0.30 × 0.30 × 0.25 mm
*V* = 1136.41 (13) Å^3^	

### Data collection

**Table:**

Bruker APEXII CCD area-detector diffractometer	5603 independent reflections
Radiation source: fine-focus sealed tube	4378 reflections with *I* > 2σ(*I*)
Graphite monochromator	*R*_int_ = 0.025
ω and φ scans	θ_max_ = 28.3°, θ_min_ = 1.5°
Absorption correction: multi-scan (*SADABS*; Bruker, 2008)	*h* = −11→11
*T*_min_ = 0.974, *T*_max_ = 0.978	*k* = −13→13
20472 measured reflections	*l* = −18→19

### Refinement

**Table:**

Refinement on *F*^2^	Primary atom site location: structure-invariant direct methods
Least-squares matrix: full	Secondary atom site location: difference Fourier map
*R*[*F*^2^ > 2σ(*F*^2^)] = 0.045	Hydrogen site location: inferred from neighbouring sites
*wR*(*F*^2^) = 0.125	H-atom parameters constrained
*S* = 1.08	*w* = 1/[σ^2^(*F*_o_^2^) + (0.0559*P*)^2^ + 0.215*P*] where *P* = (*F*_o_^2^ + 2*F*_c_^2^)/3
5603 reflections	(Δ/σ)_max_ = 0.002
319 parameters	Δρ_max_ = 0.23 e Å^−^^3^
40 restraints	Δρ_min_ = −0.22 e Å^−^^3^

### Special details

**Table:**

Geometry. All e.s.d.'s (except the e.s.d. in the dihedral angle between two l.s. planes) are estimated using the full covariance matrix. The cell e.s.d.'s are taken into account individually in the estimation of e.s.d.'s in distances, angles and torsion angles; correlations between e.s.d.'s in cell parameters are only used when they are defined by crystal symmetry. An approximate (isotropic) treatment of cell e.s.d.'s is used for estimating e.s.d.'s involving l.s. planes.
Refinement. Refinement of *F*^2^ against ALL reflections. The weighted *R*-factor *wR* and goodness of fit *S* are based on *F*^2^, conventional *R*-factors *R* are based on *F*, with *F* set to zero for negative *F*^2^. The threshold expression of *F*^2^ > σ(*F*^2^) is used only for calculating *R*-factors(gt) *etc*. and is not relevant to the choice of reflections for refinement. *R*-factors based on *F*^2^ are statistically about twice as large as those based on *F*, and *R*- factors based on ALL data will be even larger.

### Fractional atomic coordinates and isotropic or equivalent isotropic displacement parameters (Å^2^)

**Table:**

	*x*	*y*	*z*	*U*_iso_*/*U*_eq_	Occ. (<1)
C1A	−0.0430 (10)	1.0338 (8)	0.2839 (4)	0.0877 (18)	0.587 (11)
H1A1	0.0125	1.0358	0.2306	0.132*	0.587 (11)
H1A2	−0.0730	1.1174	0.2975	0.132*	0.587 (11)
H1A3	−0.1420	0.9386	0.2680	0.132*	0.587 (11)
C2A	0.0677 (12)	1.0483 (8)	0.3675 (6)	0.089 (3)	0.587 (11)
H2A1	0.0188	1.0599	0.4238	0.106*	0.587 (11)
H2A2	0.1740	1.1376	0.3795	0.106*	0.587 (11)
C1B	0.0656 (17)	1.0966 (9)	0.2980 (6)	0.088 (3)	0.413 (11)
H1B1	0.1845	1.1562	0.3183	0.131*	0.413 (11)
H1B2	0.0130	1.1639	0.3003	0.131*	0.413 (11)
H1B3	0.0393	1.0304	0.2334	0.131*	0.413 (11)
C2B	0.0054 (13)	1.0057 (11)	0.3611 (9)	0.072 (3)	0.413 (11)
H2B1	−0.1149	0.9451	0.3426	0.086*	0.413 (11)
H2B2	0.0346	1.0696	0.4274	0.086*	0.413 (11)
C3	0.21727 (18)	0.92280 (15)	0.41102 (10)	0.0423 (3)	
C4	0.26073 (15)	0.78973 (13)	0.38728 (9)	0.0328 (3)	
H4	0.3792	0.8281	0.4159	0.039*	
C5	0.15967 (15)	0.66458 (14)	0.43037 (9)	0.0346 (3)	
H5	0.0410	0.6419	0.4175	0.042*	
C6	0.22279 (18)	0.69639 (17)	0.53715 (10)	0.0466 (3)	
H6A	0.2708	0.8051	0.5713	0.056*	
H6B	0.1334	0.6416	0.5664	0.056*	
C7	0.3552 (2)	0.63749 (19)	0.53718 (11)	0.0550 (4)	
H7A	0.4601	0.7115	0.5293	0.066*	
H7B	0.3743	0.6130	0.5960	0.066*	
C8	0.2771 (2)	0.49510 (18)	0.45101 (11)	0.0503 (4)	
H8A	0.3628	0.4711	0.4251	0.060*	
H8B	0.2022	0.4082	0.4687	0.060*	
C9	0.24212 (14)	0.55059 (13)	0.28972 (9)	0.0314 (2)	
C10	0.40719 (14)	0.54634 (14)	0.28034 (9)	0.0335 (3)	
C11	0.56843 (16)	0.64768 (16)	0.32716 (11)	0.0433 (3)	
H11	0.5907	0.7389	0.3736	0.052*	
C12	0.69804 (17)	0.6109 (2)	0.30370 (12)	0.0531 (4)	
H12	0.8079	0.6793	0.3342	0.064*	
C13	0.66598 (19)	0.4754 (2)	0.23631 (12)	0.0552 (4)	
H13	0.7541	0.4521	0.2231	0.066*	
C14	0.50416 (19)	0.37281 (19)	0.18772 (11)	0.0491 (4)	
H14	0.4821	0.2810	0.1419	0.059*	
C15	0.37664 (16)	0.41162 (15)	0.20968 (9)	0.0373 (3)	
C16	0.11869 (15)	0.40657 (14)	0.20770 (9)	0.0362 (3)	
C17	0.1341 (2)	0.19559 (16)	0.08382 (11)	0.0528 (4)	
H17A	0.0135	0.1469	0.0769	0.063*	
H17B	0.1753	0.1226	0.0927	0.063*	
C18	0.1747 (2)	0.23314 (18)	−0.00835 (11)	0.0552 (4)	
H18A	0.1372	0.1376	−0.0597	0.066*	
H18B	0.2953	0.2869	0.0004	0.066*	
C19	0.0979 (2)	0.33016 (19)	−0.04075 (11)	0.0568 (4)	
H19A	0.0621	0.2915	−0.1103	0.068*	
H19B	−0.0013	0.3154	−0.0142	0.068*	
C20	0.2087 (2)	0.49895 (18)	−0.01325 (10)	0.0531 (4)	
H20A	0.3192	0.5157	−0.0248	0.064*	
H20B	0.1635	0.5445	−0.0528	0.064*	
C21	0.33250 (17)	0.72236 (15)	0.12529 (10)	0.0396 (3)	
C22	0.4290 (2)	0.80905 (19)	0.07220 (11)	0.0536 (4)	
H22	0.4166	0.7651	0.0066	0.064*	
C23	0.5431 (2)	0.95975 (19)	0.11637 (12)	0.0592 (4)	
H23	0.6082	1.0165	0.0806	0.071*	
C24	0.5606 (2)	1.02605 (18)	0.21268 (12)	0.0565 (4)	
H24	0.6379	1.1274	0.2427	0.068*	
C25	0.46165 (18)	0.94020 (16)	0.26501 (11)	0.0473 (3)	
H25	0.4736	0.9863	0.3302	0.057*	
C26	0.34589 (15)	0.78868 (14)	0.22383 (9)	0.0350 (3)	
C27	0.23201 (14)	0.69994 (13)	0.28114 (8)	0.0314 (2)	
H27	0.1165	0.6710	0.2510	0.038*	
N1	0.18398 (13)	0.53096 (12)	0.37981 (7)	0.0361 (2)	
N2	0.20527 (14)	0.32851 (12)	0.16915 (8)	0.0398 (3)	
O1	0.28603 (18)	1.03033 (14)	0.48165 (9)	0.0743 (4)	
O2	0.09278 (16)	0.90670 (14)	0.34755 (9)	0.0684 (3)	
O3	0.22133 (12)	0.57163 (11)	0.08703 (6)	0.0447 (2)	
O4	−0.03046 (11)	0.36456 (11)	0.18380 (7)	0.0494 (3)	

### Atomic displacement parameters (Å^2^)

**Table:**

	*U*^11^	*U*^22^	*U*^33^	*U*^12^	*U*^13^	*U*^23^
C1A	0.106 (4)	0.079 (3)	0.104 (3)	0.063 (3)	0.008 (3)	0.038 (3)
C2A	0.119 (6)	0.077 (4)	0.087 (4)	0.078 (4)	−0.009 (4)	0.002 (3)
C1B	0.131 (7)	0.069 (4)	0.074 (4)	0.046 (4)	0.027 (4)	0.032 (3)
C2B	0.092 (5)	0.072 (4)	0.087 (6)	0.058 (4)	0.041 (5)	0.038 (4)
C3	0.0498 (7)	0.0370 (7)	0.0411 (8)	0.0205 (6)	0.0135 (6)	0.0094 (6)
C4	0.0343 (6)	0.0329 (6)	0.0297 (6)	0.0152 (5)	0.0065 (5)	0.0064 (5)
C5	0.0346 (6)	0.0354 (6)	0.0328 (6)	0.0155 (5)	0.0097 (5)	0.0072 (5)
C6	0.0565 (8)	0.0451 (8)	0.0320 (7)	0.0163 (6)	0.0131 (6)	0.0103 (6)
C7	0.0570 (9)	0.0661 (10)	0.0420 (8)	0.0227 (8)	0.0038 (7)	0.0265 (7)
C8	0.0655 (9)	0.0601 (9)	0.0476 (9)	0.0391 (8)	0.0218 (7)	0.0304 (7)
C9	0.0315 (5)	0.0331 (6)	0.0300 (6)	0.0157 (5)	0.0067 (5)	0.0075 (5)
C10	0.0341 (6)	0.0377 (6)	0.0340 (6)	0.0193 (5)	0.0086 (5)	0.0128 (5)
C11	0.0360 (6)	0.0470 (8)	0.0474 (8)	0.0183 (6)	0.0062 (6)	0.0162 (6)
C12	0.0349 (7)	0.0702 (10)	0.0638 (10)	0.0254 (7)	0.0118 (7)	0.0319 (9)
C13	0.0528 (8)	0.0895 (12)	0.0568 (10)	0.0507 (9)	0.0274 (7)	0.0396 (9)
C14	0.0629 (9)	0.0646 (9)	0.0431 (8)	0.0460 (8)	0.0218 (7)	0.0208 (7)
C15	0.0433 (7)	0.0437 (7)	0.0347 (7)	0.0255 (6)	0.0126 (5)	0.0156 (5)
C16	0.0379 (6)	0.0353 (6)	0.0342 (7)	0.0161 (5)	0.0082 (5)	0.0081 (5)
C17	0.0673 (9)	0.0375 (7)	0.0460 (9)	0.0239 (7)	0.0076 (7)	−0.0002 (6)
C18	0.0703 (10)	0.0500 (9)	0.0403 (8)	0.0310 (8)	0.0085 (7)	−0.0018 (7)
C19	0.0667 (10)	0.0619 (10)	0.0342 (8)	0.0330 (8)	−0.0024 (7)	−0.0020 (7)
C20	0.0743 (10)	0.0599 (9)	0.0305 (7)	0.0375 (8)	0.0112 (7)	0.0090 (6)
C21	0.0473 (7)	0.0439 (7)	0.0353 (7)	0.0260 (6)	0.0094 (6)	0.0148 (6)
C22	0.0738 (10)	0.0600 (9)	0.0394 (8)	0.0345 (8)	0.0225 (7)	0.0230 (7)
C23	0.0705 (10)	0.0612 (10)	0.0576 (10)	0.0276 (8)	0.0278 (8)	0.0351 (8)
C24	0.0612 (9)	0.0471 (8)	0.0554 (10)	0.0138 (7)	0.0143 (8)	0.0226 (7)
C25	0.0530 (8)	0.0448 (8)	0.0388 (8)	0.0155 (6)	0.0103 (6)	0.0140 (6)
C26	0.0392 (6)	0.0386 (6)	0.0322 (6)	0.0201 (5)	0.0081 (5)	0.0134 (5)
C27	0.0331 (6)	0.0327 (6)	0.0287 (6)	0.0164 (5)	0.0046 (5)	0.0072 (5)
N1	0.0436 (6)	0.0373 (6)	0.0323 (6)	0.0210 (5)	0.0125 (4)	0.0115 (4)
N2	0.0451 (6)	0.0357 (6)	0.0365 (6)	0.0207 (5)	0.0071 (5)	0.0024 (4)
O1	0.0918 (9)	0.0525 (7)	0.0627 (8)	0.0368 (7)	0.0019 (7)	−0.0139 (6)
O2	0.0874 (8)	0.0622 (7)	0.0668 (8)	0.0558 (7)	−0.0023 (6)	0.0045 (6)
O3	0.0589 (6)	0.0458 (5)	0.0286 (5)	0.0243 (5)	0.0090 (4)	0.0078 (4)
O4	0.0336 (5)	0.0502 (6)	0.0515 (6)	0.0142 (4)	0.0025 (4)	0.0029 (5)

### Geometric parameters (Å, º)

**Table:**

C1A—C2A	1.443 (6)	C11—C12	1.3947 (19)
C1A—H1A1	0.9600	C11—H11	0.9300
C1A—H1A2	0.9600	C12—C13	1.373 (2)
C1A—H1A3	0.9600	C12—H12	0.9300
C2A—O2	1.487 (5)	C13—C14	1.385 (2)
C2A—H2A1	0.9700	C13—H13	0.9300
C2A—H2A2	0.9700	C14—C15	1.3827 (18)
C1B—C2B	1.438 (7)	C14—H14	0.9300
C1B—H1B1	0.9600	C15—N2	1.4011 (17)
C1B—H1B2	0.9600	C16—O4	1.2123 (15)
C1B—H1B3	0.9600	C16—N2	1.3701 (16)
C2B—O2	1.478 (6)	C17—N2	1.4524 (18)
C2B—H2B1	0.9700	C17—C18	1.521 (2)
C2B—H2B2	0.9700	C17—H17A	0.9700
C3—O1	1.1925 (18)	C17—H17B	0.9700
C3—O2	1.3145 (18)	C18—C19	1.530 (2)
C3—C4	1.5038 (17)	C18—H18A	0.9700
C4—C5	1.5333 (16)	C18—H18B	0.9700
C4—C27	1.5351 (17)	C19—C20	1.500 (2)
C4—H4	0.9800	C19—H19A	0.9700
C5—N1	1.4720 (16)	C19—H19B	0.9700
C5—C6	1.5241 (19)	C20—O3	1.4440 (16)
C5—H5	0.9800	C20—H20A	0.9700
C6—C7	1.520 (2)	C20—H20B	0.9700
C6—H6A	0.9700	C21—O3	1.3643 (16)
C6—H6B	0.9700	C21—C22	1.3930 (19)
C7—C8	1.511 (2)	C21—C26	1.4064 (18)
C7—H7A	0.9700	C22—C23	1.380 (2)
C7—H7B	0.9700	C22—H22	0.9300
C8—N1	1.4756 (17)	C23—C24	1.370 (2)
C8—H8A	0.9700	C23—H23	0.9300
C8—H8B	0.9700	C24—C25	1.3896 (19)
C9—N1	1.4867 (15)	C24—H24	0.9300
C9—C10	1.5093 (15)	C25—C26	1.3843 (19)
C9—C16	1.5442 (17)	C25—H25	0.9300
C9—C27	1.5669 (16)	C26—C27	1.5194 (16)
C10—C11	1.3734 (18)	C27—H27	0.9800
C10—C15	1.3934 (18)		
			
C1A—C2A—O2	107.4 (5)	C14—C13—H13	119.5
C1A—C2A—H2A1	110.2	C15—C14—C13	117.71 (14)
O2—C2A—H2A1	110.2	C15—C14—H14	121.1
C1A—C2A—H2A2	110.2	C13—C14—H14	121.1
O2—C2A—H2A2	110.2	C14—C15—C10	121.80 (13)
H2A1—C2A—H2A2	108.5	C14—C15—N2	127.97 (13)
C2B—C1B—H1B1	109.5	C10—C15—N2	110.23 (10)
C2B—C1B—H1B2	109.5	O4—C16—N2	125.24 (12)
H1B1—C1B—H1B2	109.5	O4—C16—C9	126.60 (11)
C2B—C1B—H1B3	109.5	N2—C16—C9	108.07 (10)
H1B1—C1B—H1B3	109.5	N2—C17—C18	113.75 (12)
H1B2—C1B—H1B3	109.5	N2—C17—H17A	108.8
C1B—C2B—O2	103.0 (6)	C18—C17—H17A	108.8
C1B—C2B—H2B1	111.2	N2—C17—H17B	108.8
O2—C2B—H2B1	111.2	C18—C17—H17B	108.8
C1B—C2B—H2B2	111.2	H17A—C17—H17B	107.7
O2—C2B—H2B2	111.2	C17—C18—C19	115.73 (13)
H2B1—C2B—H2B2	109.1	C17—C18—H18A	108.3
O1—C3—O2	123.36 (13)	C19—C18—H18A	108.3
O1—C3—C4	123.34 (14)	C17—C18—H18B	108.3
O2—C3—C4	113.27 (12)	C19—C18—H18B	108.3
C3—C4—C5	111.85 (10)	H18A—C18—H18B	107.4
C3—C4—C27	118.71 (10)	C20—C19—C18	116.20 (14)
C5—C4—C27	102.81 (9)	C20—C19—H19A	108.2
C3—C4—H4	107.6	C18—C19—H19A	108.2
C5—C4—H4	107.6	C20—C19—H19B	108.2
C27—C4—H4	107.6	C18—C19—H19B	108.2
N1—C5—C6	105.91 (10)	H19A—C19—H19B	107.4
N1—C5—C4	103.94 (9)	O3—C20—C19	110.62 (13)
C6—C5—C4	115.22 (11)	O3—C20—H20A	109.5
N1—C5—H5	110.5	C19—C20—H20A	109.5
C6—C5—H5	110.5	O3—C20—H20B	109.5
C4—C5—H5	110.5	C19—C20—H20B	109.5
C7—C6—C5	103.05 (11)	H20A—C20—H20B	108.1
C7—C6—H6A	111.2	O3—C21—C22	123.50 (13)
C5—C6—H6A	111.2	O3—C21—C26	116.04 (11)
C7—C6—H6B	111.2	C22—C21—C26	120.46 (13)
C5—C6—H6B	111.2	C23—C22—C21	120.37 (14)
H6A—C6—H6B	109.1	C23—C22—H22	119.8
C8—C7—C6	102.04 (12)	C21—C22—H22	119.8
C8—C7—H7A	111.4	C24—C23—C22	120.23 (14)
C6—C7—H7A	111.4	C24—C23—H23	119.9
C8—C7—H7B	111.4	C22—C23—H23	119.9
C6—C7—H7B	111.4	C23—C24—C25	119.26 (15)
H7A—C7—H7B	109.2	C23—C24—H24	120.4
N1—C8—C7	105.66 (11)	C25—C24—H24	120.4
N1—C8—H8A	110.6	C26—C25—C24	122.54 (14)
C7—C8—H8A	110.6	C26—C25—H25	118.7
N1—C8—H8B	110.6	C24—C25—H25	118.7
C7—C8—H8B	110.6	C25—C26—C21	117.12 (12)
H8A—C8—H8B	108.7	C25—C26—C27	121.56 (11)
N1—C9—C10	116.46 (9)	C21—C26—C27	121.25 (11)
N1—C9—C16	106.22 (9)	C26—C27—C4	114.73 (10)
C10—C9—C16	101.89 (9)	C26—C27—C9	117.37 (9)
N1—C9—C27	104.52 (9)	C4—C27—C9	101.60 (9)
C10—C9—C27	115.18 (9)	C26—C27—H27	107.5
C16—C9—C27	112.44 (9)	C4—C27—H27	107.5
C11—C10—C15	119.80 (11)	C9—C27—H27	107.5
C11—C10—C9	131.67 (12)	C5—N1—C8	108.13 (10)
C15—C10—C9	108.54 (10)	C5—N1—C9	110.23 (9)
C10—C11—C12	118.65 (14)	C8—N1—C9	120.66 (10)
C10—C11—H11	120.7	C16—N2—C15	110.85 (10)
C12—C11—H11	120.7	C16—N2—C17	123.63 (11)
C13—C12—C11	121.05 (14)	C15—N2—C17	124.23 (11)
C13—C12—H12	119.5	C3—O2—C2B	124.6 (5)
C11—C12—H12	119.5	C3—O2—C2A	111.3 (3)
C12—C13—C14	120.93 (13)	C21—O3—C20	117.33 (11)
C12—C13—H13	119.5		
			
O1—C3—C4—C5	89.09 (17)	C22—C21—C26—C27	175.06 (12)
O2—C3—C4—C5	−89.09 (14)	C25—C26—C27—C4	−2.05 (16)
O1—C3—C4—C27	−151.44 (14)	C21—C26—C27—C4	−178.96 (10)
O2—C3—C4—C27	30.38 (16)	C25—C26—C27—C9	−121.25 (13)
C3—C4—C5—N1	165.51 (10)	C21—C26—C27—C9	61.84 (15)
C27—C4—C5—N1	37.05 (11)	C3—C4—C27—C26	67.92 (14)
C3—C4—C5—C6	−79.07 (14)	C5—C4—C27—C26	−168.04 (9)
C27—C4—C5—C6	152.47 (10)	C3—C4—C27—C9	−164.39 (10)
N1—C5—C6—C7	28.37 (13)	C5—C4—C27—C9	−40.35 (11)
C4—C5—C6—C7	−85.91 (13)	N1—C9—C27—C26	155.06 (10)
C5—C6—C7—C8	−38.37 (13)	C10—C9—C27—C26	25.97 (15)
C6—C7—C8—N1	34.89 (14)	C16—C9—C27—C26	−90.16 (12)
N1—C9—C10—C11	−70.23 (17)	N1—C9—C27—C4	29.09 (11)
C16—C9—C10—C11	174.70 (13)	C10—C9—C27—C4	−99.99 (11)
C27—C9—C10—C11	52.69 (18)	C16—C9—C27—C4	143.88 (10)
N1—C9—C10—C15	110.05 (12)	C6—C5—N1—C8	−6.82 (13)
C16—C9—C10—C15	−5.02 (12)	C4—C5—N1—C8	115.00 (11)
C27—C9—C10—C15	−127.02 (11)	C6—C5—N1—C9	−140.65 (10)
C15—C10—C11—C12	−1.28 (19)	C4—C5—N1—C9	−18.83 (12)
C9—C10—C11—C12	179.03 (12)	C7—C8—N1—C5	−17.71 (14)
C10—C11—C12—C13	−1.0 (2)	C7—C8—N1—C9	110.39 (13)
C11—C12—C13—C14	1.7 (2)	C10—C9—N1—C5	121.72 (11)
C12—C13—C14—C15	−0.2 (2)	C16—C9—N1—C5	−125.67 (10)
C13—C14—C15—C10	−2.1 (2)	C27—C9—N1—C5	−6.59 (12)
C13—C14—C15—N2	178.71 (13)	C10—C9—N1—C8	−5.43 (16)
C11—C10—C15—C14	2.89 (19)	C16—C9—N1—C8	107.18 (12)
C9—C10—C15—C14	−177.35 (11)	C27—C9—N1—C8	−133.74 (11)
C11—C10—C15—N2	−177.82 (11)	O4—C16—N2—C15	177.33 (12)
C9—C10—C15—N2	1.94 (14)	C9—C16—N2—C15	−5.83 (14)
N1—C9—C16—O4	60.93 (16)	O4—C16—N2—C17	9.9 (2)
C10—C9—C16—O4	−176.69 (13)	C9—C16—N2—C17	−173.31 (12)
C27—C9—C16—O4	−52.82 (17)	C14—C15—N2—C16	−178.20 (13)
N1—C9—C16—N2	−115.86 (10)	C10—C15—N2—C16	2.56 (15)
C10—C9—C16—N2	6.52 (12)	C14—C15—N2—C17	−10.8 (2)
C27—C9—C16—N2	130.40 (10)	C10—C15—N2—C17	169.95 (12)
N2—C17—C18—C19	−66.50 (18)	C18—C17—N2—C16	96.75 (16)
C17—C18—C19—C20	97.32 (18)	C18—C17—N2—C15	−69.06 (18)
C18—C19—C20—O3	−76.86 (17)	O1—C3—O2—C2B	−9.2 (4)
O3—C21—C22—C23	−177.87 (14)	C4—C3—O2—C2B	169.0 (4)
C26—C21—C22—C23	2.2 (2)	O1—C3—O2—C2A	8.6 (5)
C21—C22—C23—C24	−0.9 (3)	C4—C3—O2—C2A	−173.2 (5)
C22—C23—C24—C25	−0.4 (3)	C1B—C2B—O2—C3	106.8 (10)
C23—C24—C25—C26	0.6 (2)	C1B—C2B—O2—C2A	52.4 (13)
C24—C25—C26—C21	0.6 (2)	C1A—C2A—O2—C3	167.2 (7)
C24—C25—C26—C27	−176.39 (13)	C1A—C2A—O2—C2B	−58.7 (15)
O3—C21—C26—C25	178.04 (12)	C22—C21—O3—C20	1.99 (19)
C22—C21—C26—C25	−1.99 (19)	C26—C21—O3—C20	−178.03 (11)
O3—C21—C26—C27	−4.92 (17)	C19—C20—O3—C21	173.01 (12)

### Hydrogen-bond geometry (Å, º)

**Table:**

*D*—H···*A*	*D*—H	H···*A*	*D*···*A*	*D*—H···*A*
C11—H11···O1^i^	0.93	2.49	3.3957 (19)	164
C12—H12···O2^ii^	0.93	2.59	3.446 (2)	153
C13—H13···O4^ii^	0.93	2.47	3.3986 (17)	175

Symmetry codes: (i) −*x*+1, −*y*+2, −*z*+1; (ii) *x*+1, *y*, *z*.
